# Ion-imprinted polymer-based sensors for toxic metal-ion detection in water: coordination chemistry, transduction strategies, and environmental applications

**DOI:** 10.3389/fchem.2026.1830143

**Published:** 2026-07-27

**Authors:** Ghita Yammouri, Gymama Slaughter

**Affiliations:** 1 Center for Bioelectronics, Old Dominion University, Norfolk, VA, United States; 2 Department of Electrical and Computer Engineering, Old Dominion University, Norfolk, VA, United States

**Keywords:** electrochemical sensors, ion-imprinted polymers, molecularly imprinted polymers, toxic metal-ion detection, water quality monitoring

## Abstract

Toxic metal contamination in aquatic environments remains a persistent threat to human health and ecosystems. Yet, the high cost, infrastructure demands, and centralized nature of conventional analytical methods constrain routine monitoring. Ion-imprinted polymers (IIPs), a subclass of molecularly imprinted polymers, have emerged as promising synthetic recognition materials for metal-ion sensing because they generate coordination-defined binding sites with high selectivity, chemical stability, low cost, and reusability. This review summarizes recent advances in Ion-imprinted polymer (IIP)-based sensing technologies for toxic metal-ion detection in water from 2016 to 2026. It examines the fundamental recognition chemistry of IIPs, major synthesis strategies used to generate selective binding cavities, and the principal sensing platforms, including electrochemical, optical/fluorescence, and piezoelectric systems. Attention is given to the role of conductive nanomaterials in enhancing electron transfer. Additional emphasis is placed on multiplex metal-detection strategies and validation in real environmental samples. Across literature, electrochemical platforms dominate the field owing to their sensitivity, portability, and compatibility with miniaturized devices, although analytical performance often depends strongly on interfacial engineering and matrix conditions. Current challenges, including reproducibility, selectivity in complex waters, and field deployment, are discussed together with future directions needed to enable practical water-quality monitoring technologies.

## 1 Introduction

Ensuring access to safe and clean water is a critical global priority due to its implications for public health, environmental sustainability, and socioeconomic development (Piwowarska et al., 2024; Roy et al., 2024). Among water contaminants, toxic metal pollution represents a persistent challenge because of its widespread occurrence and long-term environmental stability (Piwowarska et al., 2024; Saha et al., 2017). Industrial, agricultural, and urban activities introduce metal contaminants into aquatic systems, where they remain environmentally relevant even at trace concentrations (Piwowarska et al., 2024). Effective monitoring strategies are therefore essential to support water safety management and regulatory oversight (Blaszczak-Boxe et al., 2023; Karaouzas et al., 2021).

Toxic metal ions are prioritized in water quality monitoring because of the difficulty associated with their detection and quantification. In aquatic environments, metals can distribute among dissolved phases, suspended particles, sediments, and biological systems (Li et al., 2025; Mach et al., 1996). At the same time, regulatory limits are extremely low, often approaching the limits of detection (LODs) of conventional analytical techniques (Tchounwou et al., 2012). The chemical complexity of natural waters, including variable pH, ionic strength, dissolved organic matter, and competing ions, further complicates selective metal analysis. This necessitates highly sensitive and selective detection approaches capable of reliable performance in real environmental matrices (Feldmann et al., 2009; Li et al., 2025).

Conventional analytical methods, such as inductively coupled plasma mass spectrometry (ICP-MS), atomic absorption spectroscopy (AAS), and inductively coupled plasma optical emission spectroscopy (ICP-OES), remain the reference techniques for toxic metal determination due to their high sensitivity and multi-element capability (Planeta et al., 2021; Yang et al., 2021). ICP-MS offers ultra-low LODs and a wide dynamic range, but requires expensive instrumentation, centralized laboratories, and skilled personnel (Provete et al., 2024; Yang et al., 2021). As a result, their suitability for rapid, on-site, and decentralized monitoring remains limited, especially in remote or resource-limited regions where toxic metal contamination may be most severe (Abdelmonem et al., 2025). Compared with conventional laboratory-based techniques, IIP-based sensors offer a different analytical role. ICP-MS and ICP-OES provide excellent sensitivity, accuracy, and multi-element capability, making them essential for confirmatory analysis, whereas AAS is widely used for metal quantification but generally requires laboratory instrumentation and trained operators. In contrast, IIP-based sensors combine selective recognition with portable electrochemical, optical, or piezoelectric readout and are therefore attractive for rapid screening, decentralized monitoring, and potential field deployment. Although their analytical performance depends strongly on polymer design, transducer configuration, and matrix composition, they offer important advantages in terms of low cost, rapid response, simple operation, and portability. Therefore, IIP-based sensors should be considered complementary tools for screening and decentralized monitoring rather than direct replacements for reference laboratory methods (Garciá-Miranda Ferrari et al., 2020; Provete et al., 2024; Yang et al., 2021; Zheng et al., 2021).

To address these limitations, sensor-based technologies have emerged as promising tools for environmental monitoring because they enable rapid detection, portability, and potential real-time analysis (Garciá-Miranda Ferrari et al., 2020). In such systems, the recognition element plays a critical role in determining selectivity toward the target ion. However, maintaining selectivity in complex matrices remains challenging due to cross-reactivity and environmental variability (Goyal and Nigam, 2025). Although biological recognition elements have been explored due to their inherent specificity, their practical deployment is often hindered by limited stability, metal-induced denaturation, and poor long-term reproducibility under environmental conditions (Kanellis, 2018; Stortini et al., 2020).

Ion-imprinted polymers (IIPs), a subclass of molecularly imprinted polymers (MIPs), are synthesized by polymerizing functional monomers around metal-ligand complexes, followed by template removal to generate coordination-defined binding cavities. These artificial recognition sites enable selective rebinding of the target analyte while maintaining high chemical and mechanical stability (Rebelo et al., 2021). In contrast to biological receptors, MIPs exhibit resistance to pH variation, temperature changes, and organic solvents, making them well-suited for environmental sensing applications in complex aqueous matrices (Rebelo et al., 2023; Zheng et al., 2021). Because these recognition cavities originate from pre-organized metal–ligand complexes, the selectivity of IIPs is governed not only by cavity size and shape but also by the coordination geometry, donor-atom identity, ligand denticity, and metal-ion speciation during both imprinting and rebinding.

In IIPs, metal-ligand complexes are used as templates during IIP synthesis, producing coordination-specific cavities that match the size, geometry, and coordination environment of the target ion (Chen et al., 2011; Hande et al., 2015). This strategy significantly improves selectivity while reducing interference from abundant cations such as Ca^2+^, Mg^2+^, and Na^+^ in natural waters (Chen et al., 2011). Consequently, IIP-based materials have demonstrated strong analytical performance for detecting toxic metals, including Pb^2+^, Cd^2+^, Hg^2+,^ and As^3+^ at trace and ultra-trace concentrations relevant to environmental monitoring (Luo et al., 2017). The integration of IIPs with diverse sensing platforms, including electrochemical, optical, piezoelectric, and nanomaterial-based transducers, has further enhanced detection sensitivity and selectivity (Lahcen and Slaughter, 2025; Tchekwagep et al., 2022; Zarejousheghani et al., 2021). Such systems enable low detection limits, improved performance in complex matrices, and stable operation under environmental conditions, highlighting their potential as cost-effective tools for water quality monitoring (Mukendi et al., 2025; Zheng et al., 2021). Many reported IIP-based sensors already demonstrate analytical performance compatible with international drinking water standards while functioning in real water samples with minimal sample preparation (Han et al., 2024). Among these sensing approaches, electrochemical platforms have become particularly prominent due to their high sensitivity, portability, and compatibility with miniaturized devices.

This review critically examines recent advances in IIP sensors for toxic metal-ion detection in water from 2016 to 2026. Emphasis is placed on imprinting strategies, coordination-based recognition mechanisms, and integration with electrochemical, optical, and piezoelectric sensing platforms. The analytical performance of reported systems is compared with respect to sensitivity, selectivity, and applicability in real water matrices. Remaining challenges, including reproducibility, matrix interference, and field deployment, are discussed together with emerging design strategies needed to translate IIP-based sensors into practical water-quality monitoring technologies.

Several recent reviews have examined molecularly imprinted polymer (MIP) and IIP-based sensing from complementary perspectives, including environmental analysis, clean-water monitoring, priority pollutant detection, water and wastewater analysis, trace toxic metal-ion detection, portable electrochemical sensing, and water-quality monitoring (Garciá-Miranda Ferrari et al., 2020; Metwally et al., 2021; Rebelo et al., 2021; Sala et al., 2022; Tchekwagep et al., 2022; Zarejousheghani et al., 2021; Zheng et al., 2021), Additional reviews have focused on toxic metal-ion sensors in aquatic environments, IIPs for toxic metal-ion detection and wastewater treatment, MIPs as environmental separation tools, nanomaterial-based ion-imprinted electrochemical sensors, microfluidic toxic metal-ion detection, nanomaterial-enhanced trace-metal sensing, and microfluidic micro-mixing strategies for seawater analysis (Abdul Halim et al., 2024; Adhawiyah and Lee, 2026; Geng et al., 2026; Goyal and Nigam, 2025; Metwally et al., 2021; Rauf et al., 2025; Yu et al., 2022. Despite these contributions, the literature remains distributed across broad MIP sensor reviews, electrochemical platform reviews, wastewater treatment studies, environmental separation studies, microfluidic and FET-based sensing reviews, nanomaterial-enhanced sensor reviews, and toxic metal-ion monitoring reviews. Therefore, the present review is positioned as a focused and up-to-date synthesis of IIP-based sensors for toxic metal-ion detection in water from 2016 to 2026. Unlike previous reviews, it integrates coordination chemistry, imprinting strategy, synthesis method, transduction platform, nanomaterial-assisted signal enhancement, multiplex detection, analytical performance, and real-water validation, providing a practical framework for evaluating IIP sensors for rapid screening, decentralized monitoring, and potential field deployment.

## 2 Overview of IIP technology

### 2.1 Principles

**FIGURE 1:**
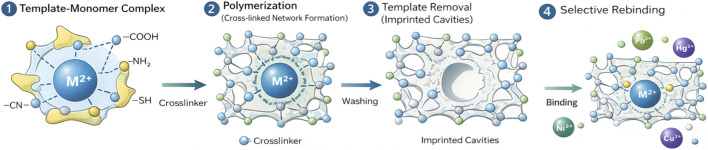
Schematic illustration of the molecular imprinting process used to produce molecularly imprinted polymers (MIPs) and ion-imprinted polymers (IIPs).

MIPs are synthetic polymer networks engineered to selectively recognize target molecules and are often described as artificial analogues of antibody-antigen systems (Animashaun and Slaughter, 2025). Molecular imprinting involves the formation of a pre-polymerization complex between a template species and functional monomers through covalent or, more commonly, noncovalent interactions such as hydrogen bonding, electrostatic forces, and π–π interactions (Piletsky and Turner, 2002). During polymerization, the monomer-template complex becomes fixed within a highly cross-linked three-dimensional (3D) matrix, typically stabilized by multifunctional crosslinkers (Animashaun and Slaughter, 2025). Subsequent removal of the template generates recognition cavities complementary in size, shape, and chemical functionality, enabling selective rebinding from complex matrices (Lahcen and Amine, 2019; Ye and Mosbach, 2008). A schematic representation of the imprinting process is shown in Figure 1.

The analytical performance of MIPs is commonly evaluated through parameters such as imprinting factor, binding capacity, and response time, which compare the behavior of imprinted and non-imprinted polymers. A key advantage of MIPs is their chemical, thermal, and mechanical robustness, allowing operation under environmental conditions unsuitable for biological receptors (Culver and Peppas, 2017). As a result, MIPs have been widely explored as recognition elements in sensing, separation, and environmental analytical technologies (L. Ye and Mosbach, 2008).

IIPs differ from organic-molecule imprinting because recognition is governed primarily by metal-ligand coordination chemistry. In IIP synthesis, the template ion forms a coordination complex with functional monomers before polymerization. The polymerization mixture typically contains functional monomers, crosslinkers, initiators, and a porogenic solvent that generates accessible porosity. Polymerization fixes the spatial arrangement of coordinating groups around the metal center, and template removal subsequently produces cavities that retain the preferred coordination geometry and donor-atom environment of the template ion (Rao et al., 2006).

In toxic metal-ion sensing applications, imprinting performance strongly depends on matching the coordination chemistry of the target ion. Soft metal ions such as Hg^2+^ and Pb^2+^ preferentially bind soft donor atoms (e.g., sulfur or nitrogen), whereas harder metal ions favor oxygen donors. Proper ligand selection, therefore, enhances binding affinity and discrimination against competing ions. Selectivity is further influenced by solution chemistry, particularly pH, which affects both proton competition for ligand sites and metal hydrolysis equilibria (Hande et al., 2015). Careful control of these parameters can significantly improve recognition performance in complex aqueous matrices.

Early studies demonstrated that polymer networks could be chemically “programmed” to selectively rebind template ions following removal, establishing the feasibility of ionic imprinting strategies (Maeda and Bartsch, 1998). Subsequent work extended these concepts to environmentally relevant metals such as Hg^2+^, enabling selective preconcentration and detection under realistic analytical conditions (Liu et al., 2005). These studies laid the foundation for the development of IIP-based materials as selective sorbents and recognition elements for environmental sensing systems.

#### 2.1.1 Coordination chemistry and metal–ligand complex formation in IIPs

Ion-imprinted polymer formation is fundamentally governed by coordination chemistry because the template metal ion first forms a pre-polymerization complex with functional monomers containing suitable donor atoms. In this process, the metal ion acts as a Lewis acid, while the functional monomer behaves as a Lewis base by donating electron density through donor atoms such as oxygen, nitrogen, sulfur, or phosphorus. The resulting metal–ligand complex defines the spatial arrangement of the coordinating groups around the template ion before polymerization. During polymerization, this arrangement is fixed within the cross-linked polymer network. Subsequent removal of the template ion leaves behind binding cavities that retain memory of the original metal-ion size, charge distribution, preferred coordination geometry, and donor-atom environment. As a result, the imprinted polymer can selectively rebind the target ion from aqueous samples through coordination interactions that partially reproduce the original pre-polymerization complex (Rao et al., 2006).

The mechanism of complex formation generally involves coordination between vacant orbitals of the metal ion and lone-pair electrons from donor atoms in the functional monomers. Monomers bearing carboxyl, hydroxyl, carbonyl, amine, pyridyl, thioether, or Schiff-base groups are commonly used because these functional groups can coordinate metal ions through O-, N-, or S-donor atoms. The stability of the pre-polymerization complex depends on several factors, including the charge and ionic radius of the metal ion, ligand denticity, donor-atom type, coordination number, geometry, pH, solvent composition, and competition from other ions. Multidentate ligands can provide stronger and more selective binding than monodentate ligands because several donor atoms interact simultaneously with the same metal ion, improving complex stability and helping preserve cavity fidelity after (Erdem et al., 2018).

The selectivity of IIPs is strongly influenced by the compatibility between the target metal ion and the donor atoms used in the imprinting process. According to hard and soft acid–base principles, harder metal ions generally favor harder donor atoms such as oxygen. In contrast, softer and more polarized metal ions preferentially interact with softer donor atoms such as sulfur or nitrogen. Therefore, ligand selection should be matched to the coordination preference of the target ion. For example, Hg^2+^ and Pb^2+^ often show strong affinity toward sulfur- and nitrogen-containing ligands, while ions such as Cd^2+^, Cu^2+^, Ni^2+^, and Cr^3+^ may interact effectively with nitrogen- or oxygen-containing functional groups depending on their coordination environment and solution chemistry. This matching between metal-ion characteristics and ligand donor atoms is essential for producing selective binding sites and reducing cross-reactivity with competing ions in natural water matrices (Ralph Pearson, 1963).

The significance of these coordination interactions extends beyond selective recognition. The strength and reversibility of metal–ligand binding determine template removal efficiency, rebinding kinetics, sensor regeneration, and long-term stability. If the metal–ligand complex is too weak, the imprinted cavity may have poor fidelity and low selectivity. Conversely, if the complex is too strong, template removal may be incomplete, or rebinding may become slow and difficult to reverse. Therefore, effective IIP design requires a balance between complex stability during polymerization and reversible rebinding during sensing. In aqueous samples, pH and metal speciation are especially important because protonation of ligand sites, hydrolysis of metal ions, and complexation with dissolved organic matter can reduce the concentration of free metal ions available for rebinding. Thus, coordination chemistry directly controls both the formation of selective recognition cavities and the analytical performance of IIP-based sensors in real water matrices (Lazar et al., 2023).

### 2.2 Synthesis methods

Multiple synthetic approaches have been developed to produce IIPs with tailored morphology, binding accessibility, and analytical performance. The polymerization strategy strongly influences site accessibility, particle morphology, and mass-transfer kinetics, all of which affect sensor performance. Thus, different preparation methods are often selected depending on whether the application involves adsorption, separation, or sensing.

#### 2.2.1 Bulk free-radical polymerization

Bulk free-radical polymerization remains one of the most widely used methods for producing IIPs due to its simplicity. In this approach, a metal-ligand complex is copolymerized with crosslinkers in the presence of a radical initiator to form a highly cross-linked polymer network. The resulting monolithic polymer is then crushed, ground, and sieved to generate particles, followed by template removal using acidic or chelating solutions (Behbahani et al., 2013). Although bulk polymerization provides high mechanical stability, grinding may damage recognition sites and generate particles with broad size distributions. In addition, many binding sites remain buried within the polymer matrix, limiting diffusion and slowing binding kinetics Cajamarca and Tarley, 2022; Pichon et al., 2020). These limitations have motivated alternative polymerization strategies that improve site accessibility.

#### 2.2.2 Precipitation polymerization

Precipitation polymerization addresses some limitations of bulk synthesis by generating particles directly without grinding. Polymerization begins in a homogeneous solution containing the template complex, monomers, crosslinkers, and initiator dissolved in a large volume of porogenic solvent. As polymer chains grow, they become insoluble and precipitate to form uniform micro- or nanoscale particles [40]. This method often produces materials with higher surface area and improved reproducibility compared with bulk polymers, although particle morphology remains sensitive to solvent composition and crosslinking conditions (Cajamarca and Tarley, 2022; Pichon et al., 2020).

#### 2.2.3 Suspension polymerization

Suspension polymerization forms spherical polymer beads by dispersing the monomer phase containing the template complex into an aqueous phase stabilized by surfactants. Each droplet acts as an independent micro-reactor in which polymerization occurs, producing mechanically stable beads suitable for packed-bed applications (Meouche et al., 2017). However, controlling droplet stability and preventing template partitioning into the continuous phase can be challenging, and particle size distributions may vary depending on agitation and stabilizer concentration.

#### 2.2.4 Emulsion and inverse-emulsion polymerization

Emulsion-based polymerization generates polymer particles within surfactant-stabilized droplets, often producing smaller particle sizes and larger interfacial areas compared with suspension systems. In inverse emulsions, polymerization occurs within water droplets dispersed in an oil phase. These approaches reduce diffusion distances and enable rapid adsorption kinetics (Li et al., 2018). However, surfactant removal can complicate purification, and particle morphology strongly depends on droplet stability during polymerization (Olsson et al., 2015; Wolska and Jalilnejad Falizi, 2021).

#### 2.2.5 Surface imprinting (core–shell architectures)

Surface imprinting strategies were developed to overcome mass-transfer limitations associated with bulk imprinting. In these systems, a thin imprinted polymer layer is formed on a supporting material to create a core-shell architecture in which recognition sites are located primarily near the surface. This configuration enhances template accessibility, rebinding kinetics, and template removal efficiency (Li et al., 2018). Surface-imprinted materials are particularly attractive for sensing applications because recognition sites are directly exposed to the analyte solution. However, synthesis typically requires additional support preparation and careful control of film thickness to avoid diffusion limitations (Guadaño-Sánchez et al., 2024; Radfar et al., 2024).

#### 2.2.6 Electropolymerization (electro-synthesized IIP films)

Electropolymerization provides a powerful route for generating thin imprinted films directly on electrode surfaces. Polymerization is initiated electrochemically in a solution containing electropolymerizable monomers and the template ion, allowing precise control of film thickness through applied potential or deposition time (Fafa and Zazoua, 2024). Because the resulting films are thin and integrated with the sensing interface, electropolymerized IIPs often exhibit rapid mass transport and high analytical sensitivity. However, this approach is limited to monomers capable of electrochemical polymerization, and film stability may depend strongly on deposition conditions (Ahmad et al., 2019; Ramanavičius et al., 2022).

Overall, IIP synthesis strategies involve trade-offs between synthetic simplicity, particle uniformity, and binding-site accessibility. Bulk and suspension polymerization remain attractive for large-scale sorbent production, whereas precipitation polymerization, surface imprinting, and electropolymerization are increasingly favored for sensing applications because they generate surface-accessible recognition sites and improved mass-transfer kinetics. Table 1 summarizes the principal advantages and limitations of common IIP preparation approaches.

**TABLE 1:** Common IIP synthesis strategies used in toxic metal-ion sensing and separation.

Method	Key advantages	Key limitations	Ref.
Bulk free-radical polymerization	Simple synthesis; high crosslinking and stability; flexible monomer design	Grinding required; broad particle size; many buried sites → slower kinetics	Behbahani e t al. (2013)
Precipitation polymerization	No grinding; relatively uniform particles; higher surface area	Large solvent volume required; particle morphology sensitive to solvent conditions	Murat et al. (2022), Zhang et al. (2022)
Suspension polymerization	Direct bead formation; tunable bead size; mechanically robust particles	Requires stable droplet dispersion; possible template partitioning; broader size distribution	Chaudhary and Sharma (2019), Meouche et al. (2017)
Emulsion/inverse-emulsion polymerization	Small particles; high interfacial area; rapid adsorption/desorption kinetics	Surfactant removal required; synthesis conditions strongly affect reproducibility	Fafa and Zazoua (2024), Li et al. (2018), Olsson et al. (2015)
Surface imprinting (core–shell; magnetic supports)	Surface-accessible sites; rapid binding and elution; effective for sensors and solid-phase extraction	Multi-step synthesis; film thickness must be controlled; support materials increase complexity	Guadaño-Sánchez et al. (2024), Radfar et al. (2024), Wei et al. (2020)
Electropolymerization (thin IIP films)	Direct electrode integration; controlled film thickness; fast response; suitable for miniaturized sensors	Limited monomer selection; possible film fouling/delamination; small sorbent capacity	Chaudhary and Sharma (2019), Fafa and Zazoua (2024)

From a sensing perspective, the choice of synthesis method strongly determines binding-site accessibility, response time, and reproducibility. Bulk polymerization provides mechanically stable materials and simple preparation, but many recognition sites remain buried inside the polymer network, leading to slower mass transfer and reduced accessibility of hydrated metal ions. Precipitation and emulsion polymerization generate smaller particles with higher surface area, which can improve rebinding kinetics; however, their morphology and reproducibility depend strongly on solvent composition, surfactant use, and polymerization conditions. Surface imprinting and electropolymerization are generally more suitable for sensor fabrication because they localize recognition sites near the transducer surface, reduce diffusion barriers, and improve signal coupling. Nevertheless, these methods require careful control of film thickness because overly thick films can block ion transport and increase resistance, whereas very thin films may reduce binding capacity. Therefore, IIP synthesis should be selected according to the intended sensing format, balancing synthetic simplicity, binding-site accessibility, mechanical stability, and compatibility with the transducer interface (Cajamarca and Tarley, 2022; Ramanavičius et al., 2022; Sala et al., 2022).

## 3 Sensor platforms, configurations, and transduction strategies

(Chaudhary and Sharma, 2019) IIPs have been integrated with diverse sensing platforms for selective detection of toxic metal ions in aqueous systems. In these sensors, analytical performance depends not only on imprinting chemistry but also on how the recognition layer interfaces with the transducer and how binding events are converted into measurable signals. Consequently, sensor architecture, including polymer configuration, interfacial design, and transduction strategy, strongly influences sensitivity, selectivity, and response kinetics. Among reported configurations, electrochemical sensors dominate the field because of their compatibility with aqueous media, high sensitivity, and straightforward miniaturization. Optical and fluorescence approaches provide alternative signal generation based on light-matter interactions, while piezoelectric devices depend on mass-sensitive transduction. Each architecture represents a different strategy for translating coordination-driven recognition within IIP cavities into measurable analytical responses.

The sensing mechanism in IIP-based platforms depends on the coupling between selective ion rebinding and the transduction process. In electrochemical sensors, rebinding of the target ion within imprinted cavities changes interfacial charge transfer, redox current, impedance, or stripping response. In optical and fluorescence sensors, ion rebinding modifies absorbance, fluorescence intensity, refractive index, or plasmonic response. In piezoelectric sensors, selective binding increases the mass of the sensing layer and produces a measurable frequency shift. Therefore, analytical performance is not controlled only by the selectivity of the imprinted cavities, but also by how efficiently the transducer converts ion-recognition events into measurable signals. This explains why sensors with similar imprinting chemistry may show different sensitivities, response times, and limits of detection depending on the selected transduction strategy (Diltemiz et al., 2017; Huang et al., 2023; Rebelo et al., 2023; Sala et al., 2022).

### 3.1 Electrochemical sensors

Electrochemical platforms represent the most mature class of IIP-based toxic metal-ion sensors because they enable sensitive detection in aqueous media while allowing direct integration with miniaturized electrodes (Faysal et al., 2025). In these systems, the IIP layer acts as a selective coordination interface that modulates electron transfer or mass transport at the electrode surface. A common implementation involves coating a glassy carbon electrode with an IIP film immobilized using a polymeric binder (Hu et al., 2016). Metal ions selectively rebind within the polymer matrix, altering the interfacial electrochemical response typically measured by voltammetry. Although this architecture offers simple fabrication, diffusion limitations within the polymer film and increased electrical resistance from binders can reduce sensitivity.

**FIGURE 2:**
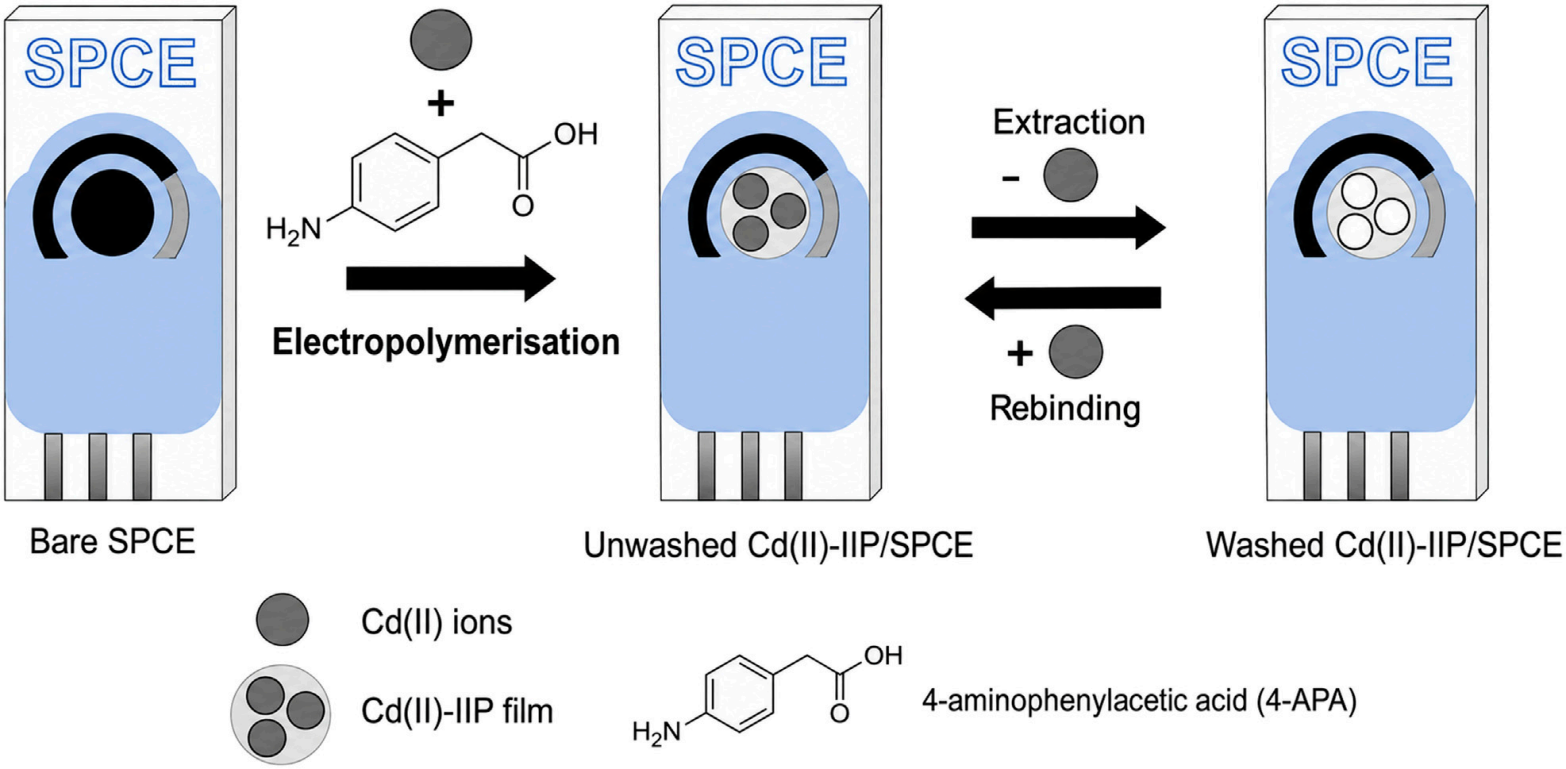
Representative electrochemical architectures for IIP-based toxic metal-ion sensing. Fabrication and sensing mechanism of a disposable SPCE modified with a Cd^2+^ ion-imprinted polymer (Cd^2+^-IIP) (Costa et al., 2023).

Multilayer designs incorporating conductive intermediate layers have therefore been developed. For example, Velempini et al. electropolymerized a polypyrrole layer on a glassy carbon electrode before depositing sulfur-containing IIP particles for Hg^2+^ detection (Velempini et al., 2018). The conducting polymer improves adhesion and electron transport, whereas the imprinted particles provide selective binding sites. Since multilayer interfaces may increase charge-transfer resistance and hinder analyte diffusion, a more integrated design using a screen-printed carbon electrode (SPCE) with electropolymerized IIP films (Figure 2) has been developed (Costa et al., 2023). In this approach, the polymer layer is grown directly on the electrode surface from a monomer-template solution. Template removal produces surface-accessible coordination cavities that are electrically coupled to the electrode, eliminating binders and improving sensitivity.

Oseyemi and Rezai demonstrated an advanced configurations integrating IIPs into microfluidic electrochemical systems as illustrated in Figure 2 (Oseyemi and Rezai, 2025b). In these devices, the imprinted polymer is implemented as a membrane within a flow-through chamber positioned between planar electrodes. Controlled fluid flow enhances mass transport and reduces sample volume requirements. However, microfluidic architectures introduce additional engineering complexity and may be susceptible to fouling in environmental samples. Overall, electrochemical IIP sensors provide strong analytical performance through direct coupling of coordination-based recognition with sensitive electrochemical readout mechanisms. Achieving optimal performance requires balancing film thickness, binding-site accessibility, and interfacial conductivity.

### 3.2 Optical and fluorescence sensors

Optical sensors represent another important class of IIP-based detection platforms because they enable non-electrical signal transduction and often allow simple visual or spectroscopic readout (Huang et al., 2023). In these systems, analyte binding within the imprinted polymer alters optical properties such as absorbance, reflection, or refractive index. One common configuration involves optical fiber sensors, where a surface-imprinted polymer layer is formed on a quartz or glass fiber (Meza López et al., 2021). The binding of the target ion modifies the refractive index or optical absorption at the sensing interface, enabling remote detection through transmitted or reflected light. Although these sensors are compact and suitable for *in-situ* monitoring, surface fouling and polymer degradation can limit long-term stability.

**FIGURE 3:**
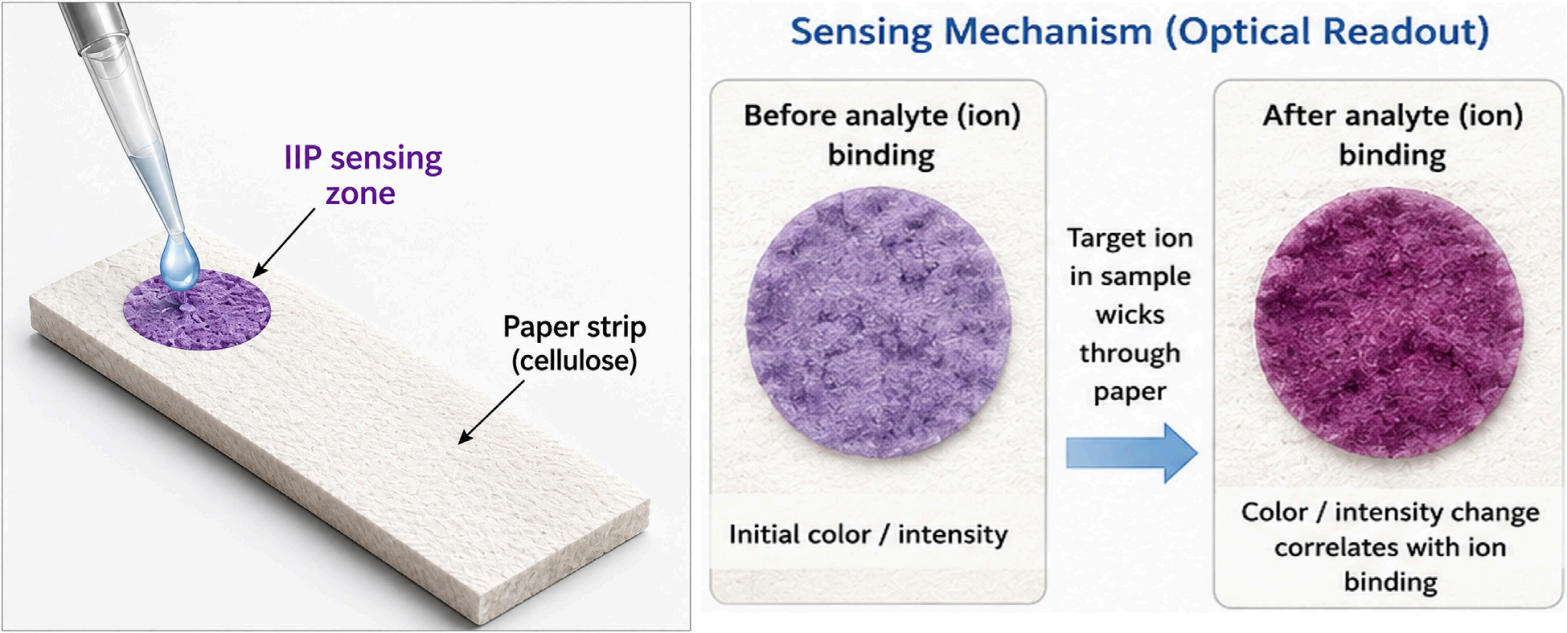
Operating principle of a paper-based optical IIP sensor. An IIP sensing layer is formed on a cellulose paper substrate, which provides both mechanical support and capillary-driven sample transport. Selective binding of target ions to the imprinted cavities results in a detectable change in color intensity, enabling low-cost optical ion sensing.

Simpler formats use planar or particulate IIP coatings on transparent substrates, in which metal-ion binding induces a visible color change or a spectroscopic absorbance change (Qi et al., 2023). These systems emphasize low-cost fabrication and straightforward readout but are often limited by diffusion-controlled transport within the polymer layer. As shown in Figure 3, paper-based optical sensors provide an alternative low-cost strategy (Huang et al., 2017). In these devices, IIPs are formed on cellulose fibers, where the paper acts as both structural support and fluid-transport medium. Capillary flow enables passive sample transport while colorimetric changes within the imprinted layer generate the analytical signal. Although attractive for portable monitoring, these devices may suffer from mechanical fragility and dimensional instability during prolonged exposure to aqueous samples.

Fluorescence-based IIP sensors provide higher analytical sensitivity by coupling imprint-based recognition with fluorescence modulation (De Acha et al., 2019; René et al., 2020). Particle-based fluorescent IIPs prepared by precipitation polymerization incorporate fluorophores within the polymer matrix, and metal-ion binding alters emission intensity. While these systems offer rapid response due to surface-accessible binding sites, stable optical measurements in turbid environmental samples remain challenging. More complex architectures, combine polydopamine-coated substrates with aptamer-assisted imprinting (Ye et al., 2025). In these sensors, a thin polydopamine film forms surface-accessible recognition cavities while fluorescence signals are generated at the solid-liquid interface. Although these designs provide high sensitivity, their reliance on noble-metal substrates and complex fabrication can limit scalability.

### 3.3 Piezoelectric sensors

Piezoelectric transducers, particularly quartz crystal microbalance (QCM) devices, represent another platform for IIP-based metal detection (Diltemiz et al., 2017). In QCM sensors, analyte binding shifts the resonance frequency of a vibrating quartz crystal due to mass accumulation on the sensing surface. Typically, a thin IIP film is immobilized on the gold-coated surface of the quartz crystal [66]. Metal-ion rebinding increases the mass of the sensing layer, producing a frequency shift proportional to analyte concentration. Because the signal arises from mass changes rather than optical or electrochemical processes, QCM sensors enable label-free detection and can operate effectively in aqueous environments. However, analytical performance depends strongly on the mechanical stability and adhesion of the imprinted polymer film, which must withstand repeated swelling and deswelling during operation. In addition, QCM systems require controlled flow conditions and vibration isolation, which can complicate miniaturization for field deployment.

Overall, electrochemical IIP sensors currently offer the most favorable balance between sensitivity, portability, and compatibility with aqueous samples, especially when stripping voltammetry or differential pulse techniques are used. Their high sensitivity arises from the combination of selective preconcentration within imprinted cavities and efficient electrochemical signal amplification at the electrode surface. Optical and fluorescence sensors provide simpler or non-electrical readout and are attractive for disposable, visual, or remote sensing formats, but their performance can be affected by turbidity, background color, fluorescence quenching, and optical scattering in real waters. Piezoelectric sensors enable label-free detection based on mass changes, but they require mechanically stable films, controlled flow conditions, and reduced environmental vibration. Thus, platform selection should be guided by the intended application: electrochemical sensors are best suited for trace quantification, optical systems for rapid field screening, and piezoelectric devices for label-free monitoring under controlled (Diltemiz et al., 2017; Huang et al., 2023; Sala et al., 2022).

## 4 Types of toxic metal ions detected using IIP-Based sensors

IIP sensors have been widely applied for selective detection of toxic metal ions in water, with imprinting strategies tailored to the coordination chemistry and speciation behavior of individual metals. In these systems, the analytical response arises from rebinding of the target ion within cavities whose geometry and performance depend strongly on the compatibility between the imprinted coordination environment and the target ion’s coordination preferences. IIP sensors have been reported for both single-metal detection and simultaneous multi-metal monitoring (Ahmad and Bhattacharya, 2019; Aravind and Mathew, 2018; Bjørklund et al., 2017; Di Masi et al., 2020; Ding et al., 2025; Fadillah et al., 2022; Fafa and Zazoua, 2024; Fitzgerald, 1998; Hossini et al., 2022; Hu et al., 2019; Hu et al., 2024; Ma et al., 2020; Meza López et al., 2021; Mohammed et al., 2023; Oseyemi and Rezai, 2025a; Prueitt et al., 2020; Qiu et al., 2026; Rebolledo-Perales et al., 2022; Sadiq et al., 2023; Shamsipur et al., 2018; Wang et al., 2021a; Wang et al., 2021b; Wu et al., 2018; Zhiani et al., 2016; Zhou et al., 2020). The following sections summarize representative systems for key environmentally relevant metals, with attention to analytical performance, preferred coordination environments, donor-atom interactions, and matrix-dependent speciation behavior.

### 4.1 Cadmium (Cd^2+^)

**FIGURE 4:**
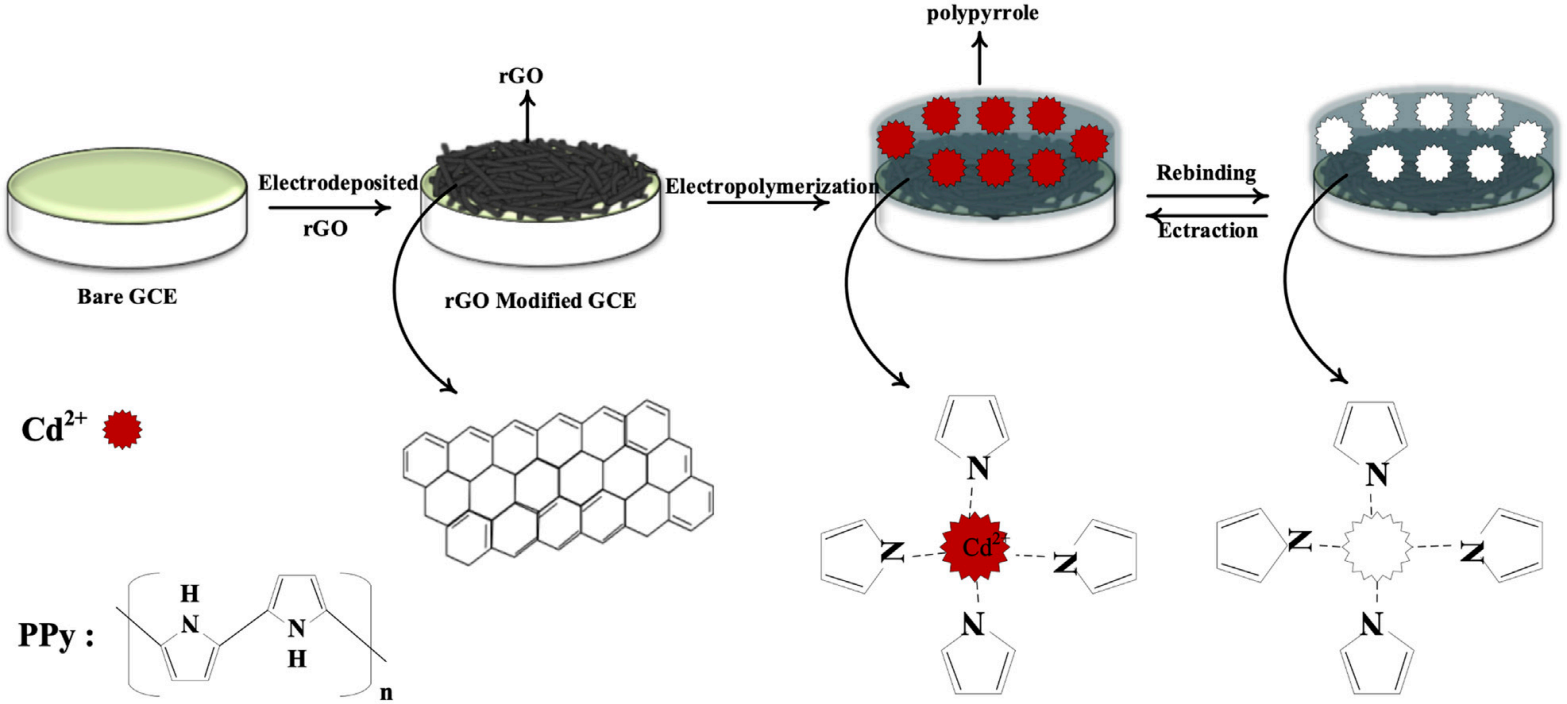
Representative IIP-based sensor platforms for Cd^2+^ and Pb^2+^ detection. Electrochemical Cd^2+^ sensor based on a polypyrrole ion-imprinted layer deposited on an reduced graphene oxide (rGO)-modified glassy carbon electrode (Hu et al., 2019).

Cadmium is a priority environmental contaminant due to its high toxicity, potential for bioaccumulation, and long biological half-life (Wang et al., 2021). Cd^2+^ typically forms stable complexes with nitrogen- and sulfur-donor ligands, making these functional groups suitable for imprinting strategies. Hu et al. reported an electrochemical sensor based on a polypyrrole/reduced graphene oxide composite IIP in which Cd^2+^ was coordinated with nitrogen-containing functional groups during polymerization (Hu et al., 2019). After template removal (Figure 4), selective Cd-N rebinding enabled detection using square-wave anodic stripping voltammetry (SWASV) with a detection limit of 0.26 μg L^-1^ and a linear range of 1–100 μg L^-1^. Minimal interference from Mg^2+^, Zn^2+^, Mn^2+^, and Cr species demonstrated effective coordination matching.

From a coordination perspective, Cd^2+^ is a borderline-soft divalent ion that can interact with N-, S-, and O-donor ligands; therefore, monomers containing amine, pyridyl, thiol, or carboxyl groups are often selected to create Cd^2+^-compatible binding sites.

An impedance-based electrochemical sensor developed by Sadiq et al. used oxygen-donor ligands within the polymer matrix to generate Cd^2+^-specific cavities (Sadiq et al., 2023). Although the system showed good selectivity against Zn^2+^, Cu^2+^, Pb^2+^, and Hg^2+^, its detection limit (∼0.10 mg L^-1^) was significantly higher than that of voltammetric systems, highlighting the influence of transduction strategy on sensitivity. More recently, Fafa and Zazoua reported an electropolymerized PEDOT-based IIP film on a screen-printed electrode incorporating sulfur- and oxygen-donor ligands (Fafa and Zazoua, 2024). The resulting sensor achieved a detection limit of 0.07 μg L^-1^ over a range of 0.5–75 μg L^-1^, reflecting improved ligand coordination and direct electrode integration. However, Cd^2+^ detection remains sensitive to solution chemistry, particularly hydrolysis and complexation equilibria.

### 4.2 Lead (Pb^2+^)

Lead is one of the most widely studied targets in IIP sensing because of its severe neurotoxicity and extremely low regulatory limits in drinking water (Meza López et al., 2021). Pb^2+^ exhibits flexible coordination geometries and strong affinity for nitrogen- and oxygen-donor ligands. Shamsipur et al. developed a Pb^2+^-imprinted nanoparticle incorporating terpyridine ligands that form strong Pb–N complexes (Shamsipur et al., 2018). Coupled with differential pulse anodic stripping voltammetry (DPASV), the sensor achieved a detection limit of approximately 0.1 nM with strong selectivity against Cd^2+^, Cu^2+^, Zn^2+^, and Ni^2+^.

Optical sensing strategies have also been explored. Meza López et al. reported an optical fiber IIP sensor based on Pb-O coordination interactions (Figure 4), enabling reflectance-based detection with a limit of detection (LOD) of 85 ng L^-1^ (Meza López et al., 2021). Although optical systems offer simpler instrumentation, their sensitivity is generally lower than that of electrochemical stripping techniques. Additional electrochemical sensors employing carboxylate and amine functional groups have achieved sub-ppb sensitivity with minimal interference from Cu^2+^ and Fe^3+^ (Mohammed et al., 2023). Microfluidic integration has also been demonstrated using Pb^2+^-imprinted membranes incorporated into flow-based electrochemical devices (Oseyemi and Rezai, 2025a). However, Pb^2+^ speciation and complexation with dissolved organic matter remain key factors influencing sensor performance in natural waters.

The relatively flexible coordination sphere of Pb^2+^ allows interaction with multiple donor atoms, but stronger and more selective recognition is generally achieved when the imprinted cavity preserves both the appropriate donor-atom arrangement and the steric environment around the Pb^2+^ template.

### 4.3 Chromium and mercury

**FIGURE 5:**
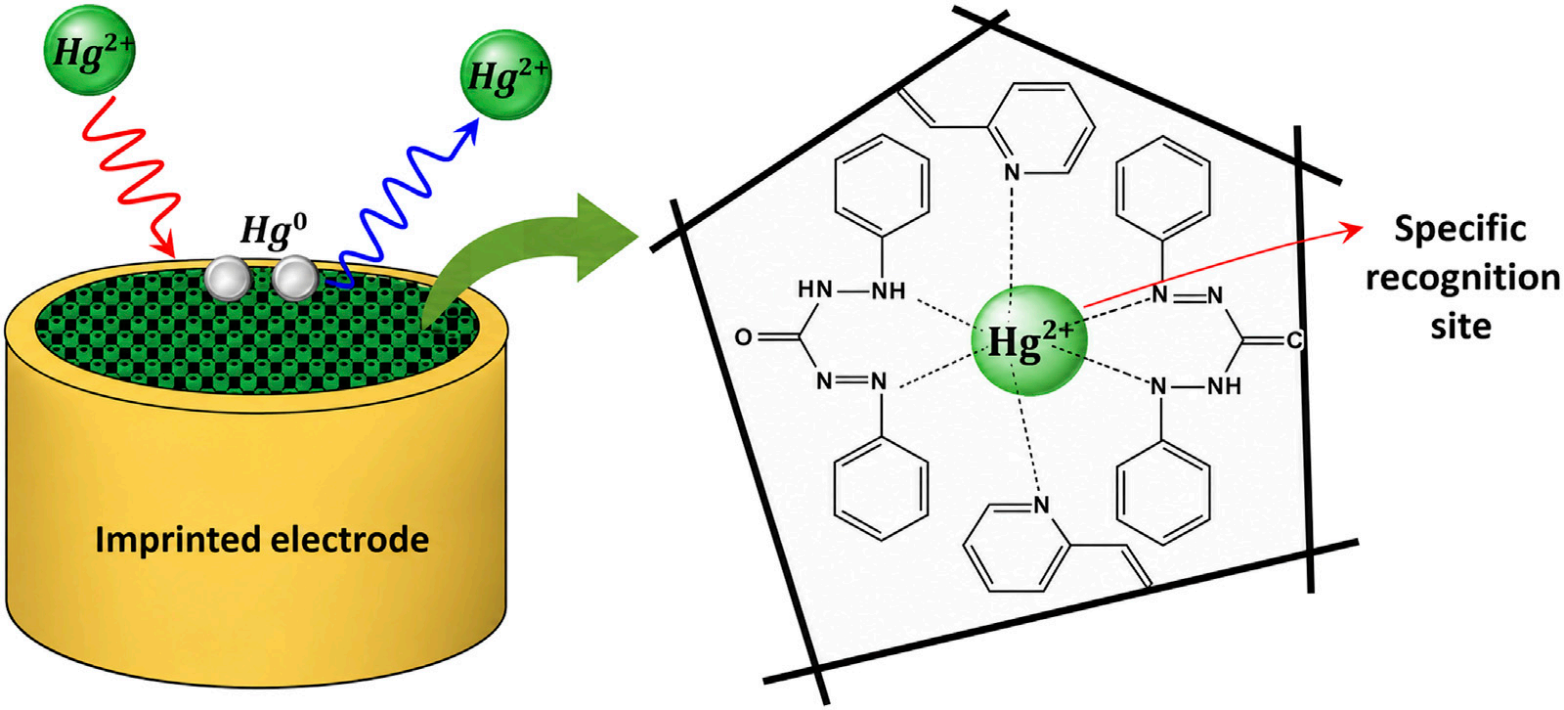
IIP fabrication strategies and sensing mechanisms for toxic metal-ion detection. Electrochemical detection mechanism based on selective Hg^2+^ rebinding at imprinted recognition sites (Fadillah et al., 2022).

Chromium presents a unique sensing challenge because it exists primarily as Cr^3+^ and Cr^6+^, which exhibit distinct chemical behaviors and toxicological profiles (Hossini et al., 2022). For Cr^3+^ detection, Aravind and Mathew developed an IIP sensor utilizing carboxylate-based coordination with trivalent chromium (Aravind and Mathew, 2018). The system enabled differential pulse voltammetric (DPV) detection with a limit of approximately 0.05 µM, although performance was affected by chromium hydrolysis near neutral pH. For Cr^6+^ detection, Wu et al. reported an electrodeposited chitosan-graphene IIP sensor (Figure 5), achieving an ultra-low detection limit of 6.4 × 10^−10^ M using DPV (Wu et al., 2018). Improved sensitivity partly reflects the higher solubility and mobility of hexavalent chromium species.

For chromium species, coordination chemistry is complicated by oxidation state and aqueous speciation because Cr^3+^ behaves as a cationic species capable of coordinating with oxygen- or nitrogen-donor ligands, whereas Cr^6+^ is commonly present as oxyanion forms under many aqueous conditions.

Mercury is another critical contaminant due to its extreme toxicity and bioaccumulation potential (Bjørklund et al., 2017). Hg^2+^ behaves as a soft Lewis acid with strong affinity for sulfur- and nitrogen-donor ligands. Rebolledo-Perales et al. reported a pyrrole-based IIP sensor in which Hg^2+^ recognition occurred through nitrogen coordination interactions (Figure 5) (Rebolledo-Perales et al., 2022). Using anodic stripping voltammetry (ASV), the sensor achieved a detection limit of 0.02 mg L^-1^ with high selectivity against Cd^2+^, Pb^2+^, and Cu^2+^. A related system using sulfur-containing ligands strengthened Hg^2+^ binding within imprinted cavities (Figure 5) and achieved a detection limit near 70 nM (Fadillah et al., 2022). Nevertheless, Hg^2+^ detection remains sensitive to pH-dependent hydrolysis and complexation with natural ligands in environmental waters.

Consequently, sulfur-containing ligands can improve Hg^2+^ recognition because soft donor atoms form stronger interactions with soft, polarizable metal centers, increasing cavity selectivity toward mercury over harder competing ions.

### 4.4 Other toxic metal ions

IIP sensors have also been developed for metals such as Cu^2+^, Ni^2+^, Ag^+^, and As^3+^. These systems illustrate the adaptability of imprinting chemistry to different coordination preferences and environmental monitoring needs (Fitzgerald, 1998). Di Masi et al. reported a Cu^2+^-imprinted electrochemical sensor based on amine coordination with a detection limit of 2.7 nM (Di Masi et al., 2020). Although the sensor maintains selectivity against competing ions, Cu^2+^ speciation and complexation in natural waters may influence analytical accuracy (Prueitt et al., 2020).

Nickel detection has been demonstrated using Ni^2+^-imprinted chitosan materials integrated with optical fiber sensors, achieving LOD near 1 nM (Qiu et al., 2026). However, signal attenuation in natural waters was observed due to matrix effects such as dissolved organic matter and ionic strength. In addition, silver detection has been achieved using Ag^+^-imprinted polymers containing sulfur- and nitrogen-donor ligands, enabling nanomolar detection using differential pulse stripping voltammetry (DPSV) (Ding et al., 2025; Zhiani et al., 2016). Similarly, arsenic detection has been reported using As^3+^-imprinted polymers integrated with nanoporous gold electrodes, achieving LOD near 7 × 10^−12^ M (Ma et al., 2020). In both cases, the analytical response depends strongly on metal speciation and solution chemistry (Ahmad and Bhattacharya, 2019).

These examples further demonstrate that IIP design should be metal-specific: Cu^2+^ and Ni^2+^ commonly benefit from N- and O-donor coordination environments, Ag^+^ shows strong affinity for softer sulfur- or nitrogen-containing ligands, and As^3+^ detection requires careful consideration of hydrolysis and aqueous speciation.

### 4.5 Simultaneous detection of multiple toxic metal ions

Although most IIP sensors target individual metals, recent studies have explored multi-metal sensing systems to address the complexity of environmental contamination. Zhou et al. reported a dual-metal fluorescent sensor based on ZnSe quantum dots (Figure 5) that enabled simultaneous detection of Cd^2+^ and Pb^2+^ with LODs of 0.245 μg L^-1^ and 0.335 μg L^-1^, respectively (Zhou et al., 2020). However, differences in coordination strength can influence binding equilibria under varying pH conditions. More complex systems capable of detecting Cu^2+^, Cd^2+^, Pb^2+^, and Hg^2+^ simultaneously have also been reported, with fluorescence-based LODs as low as 0.007–0.015 μg L^-1^ (Wang et al., 2021b). Similarly, Hu et al. developed a dual-ion imprinted microfiber sensor array for simultaneous Cr^3+^ and Ni^2+^ detection using optical wavelength-shift measurements (Hu et al., 2024). Although sub-nanomolar sensitivity was achieved, slower binding kinetics for Cr^3+^ were observed due to strong hydration effects.

According to reported studies, Pb^2+^ and Cd^2+^ sensors typically achieve the lowest LODs due to favorable coordination kinetics and strong electrochemical stripping responses. In contrast, sensing of metals such as Cr^3+^ and Hg^2+^ is more strongly influenced by hydrolysis, hydration effects, and complexation equilibria, which can reduce the concentration of free ions available for rebinding. Collectively, these studies demonstrate that IIP sensors provide a versatile platform for toxic metal-ion detection. However, analytical performance remains strongly influenced by metal coordination chemistry, aqueous speciation, and matrix effects, which remain critical considerations for environmental monitoring applications.

## 5 Role of nanomaterials in IIP-Based toxic metal-ion sensors

Integration of nanomaterials into IIP sensors has become a key strategy for overcoming intrinsic limitations of polymeric recognition layers, particularly low electrical conductivity, restricted mass transport, and diffusion-limited binding kinetics in aqueous media. In most architectures, nanomaterials do not provide chemical selectivity; instead, they function as interfacial transduction platforms that amplify optical or electrochemical signals generated by metal-ion rebinding within imprinted cavities. Thus, the analytical performance of hybrid IIP sensors depends strongly on the physicochemical compatibility between the nanomaterial scaffold and the imprinted polymer matrix.

Therefore, the coordination-defined IIP cavity remains the primary source of molecular selectivity, whereas nanomaterials mainly improve signal transduction, electron transfer, mass transport, and accessibility of the recognition sites.

Nanomaterials enhance sensor performance through several complementary mechanisms. Conductive nanostructures such as graphene derivatives and carbon nanotubes establish efficient electron-transfer pathways that compensate for the insulating character of polymer films. Semiconductor nanomaterials, including quantum dots, support fluorescence or photoelectrochemical transduction, and nanostructured supports increase the fraction of imprinted sites accessible to hydrated metal ions, thereby improving binding kinetics and sensitivity (Lahcen and Slaughter, 2025). Structurally, nanomaterials are typically integrated as conductive underlayers, dispersed composite fillers, or nanostructured carriers on which imprinting occurs directly. Each configuration affects cavity accessibility, interfacial charge transport, and long-term stability in aqueous systems.

### 5.1 Graphene family

Graphene and its derivatives are among the most extensively studied nanomaterials in IIP-based toxic metal-ion sensors because their two-dimensional sp2-carbon structure combines high carrier mobility, large surface area, and good compatibility with polymer matrices. Graphene oxide (GO), rGO, electrochemically reduced graphene oxide (ERGO), and 3D porous graphene frameworks are the most common forms used in these systems. GO is often preferred in aqueous IIP sensors because oxygen-containing functional groups improve dispersibility and compatibility with hydrophilic polymers (Jiříčková et al., 2022). For example, a chitosan/GO Cu^2+−^imprinted sensor used GO to increase the effective surface area and maintain accessibility of imprinted cavities under hydrated conditions (Wei et al., 2019). However, excessive GO loading may compromise electrical communication with the electrode.

**FIGURE 6:**
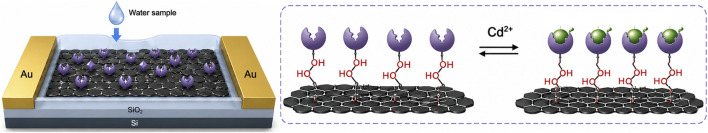
Schematic illustration of a chemiresistive IIP/rGO sensor for Cd^2+^ detection. An ion-imprinted polymer is immobilized on an rGO sensing layer between Au electrodes on a SiO_2_/Si substrate. Upon exposure to a water sample, Cd^2+^ ions selectively bind to the imprinted cavities, altering the electrical conductivity of the rGO channel and generating a chemiresistive signal proportional to the Cd^2+^ concentration.

By contrast, rGO restores much of graphene’s conductivity while retaining enough surface functionality for polymer anchoring (Rowley-Neale et al., 2018). In a chemiresistive Cd^2+^ sensor based on rGO functionalized with an IIP (Figure 6), selective Cd^2+^ rebinding induced changes in channel conductivity, enabling sensitive detection in water (Hu et al., 2021). In this configuration, the imprinted polymer provides molecular selectivity, whereas rGO converts interfacial charge perturbations into an electrical signal. However, nonspecific adsorption and ionic screening at the graphene-solution interface can attenuate output.

ERGO is frequently generated directly on electrode surfaces, producing highly conductive films with strong electrical contact (Boontanon et al., 2023). This strategy has been used in Cd^2+^ sensors based on ERGO-supported poly (o-phenylenediamine) imprinted layers, where the graphene substrate improved signal transfer during rebinding (Wang et al., 2020). Nonetheless, graphene restacking and polymer overgrowth may reduce electroactive areas during repeated sensing cycles. Similarly, 3D porous graphene architectures further enhance electrolyte penetration and mass transport (Jha et al., 2025). A Cd^2+^ sensor employing a 3D porous rGO scaffold showed how interconnected conductive frameworks can improve accessibility of imprinted sites and accelerate ion diffusion through the network (Wang et al., 2023). However, pore blockage and fouling remain important concerns in complex environmental waters.

### 5.2 Gold nanoparticles

**FIGURE 7:**
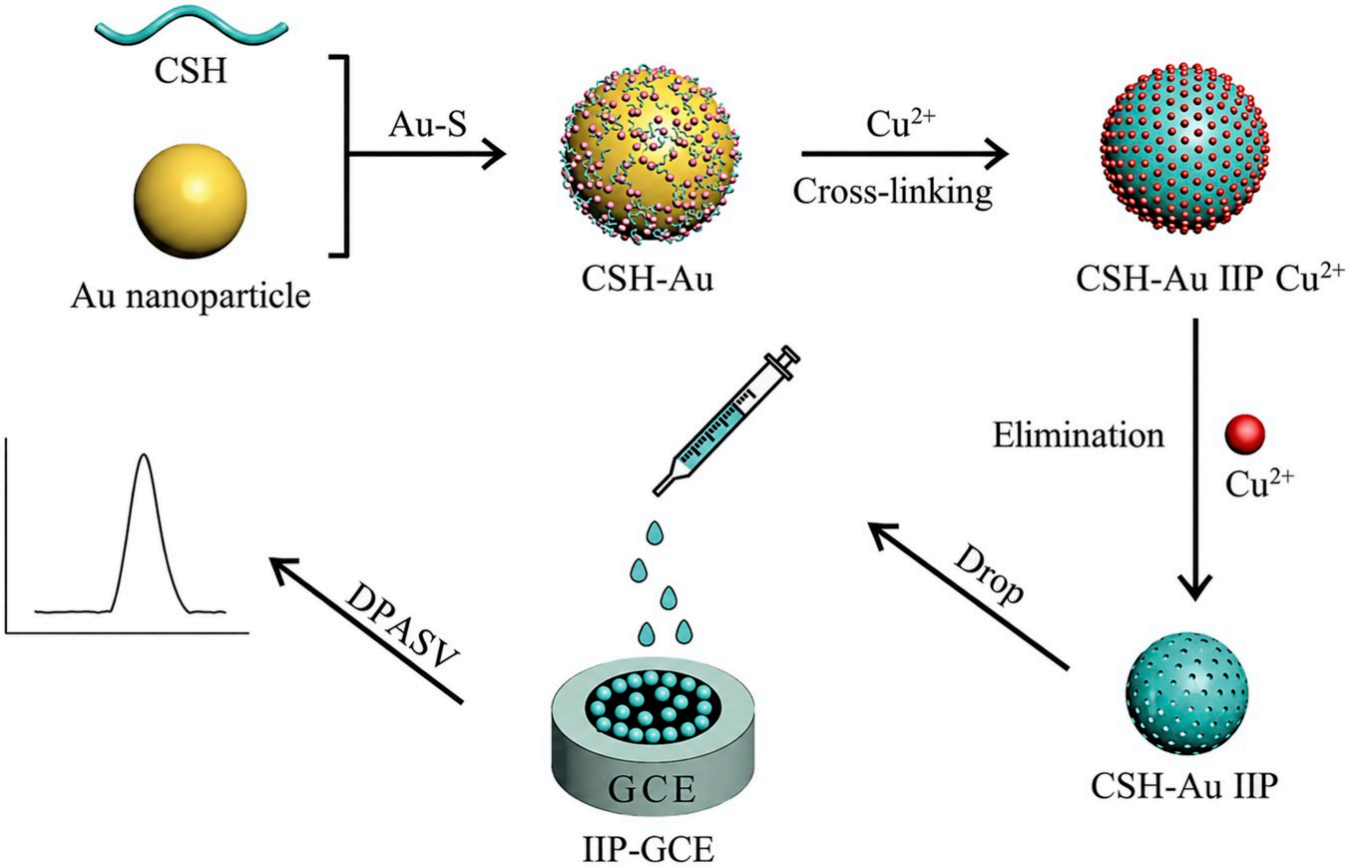
Schematic of the fabrication and sensing mechanism of a Cu^2+^-IIP based on AuNPs. CSH-functionalized AuNPs are cross-linked with Cu^2+^ to form the imprinted polymer, followed by template removal. The resulting IIP is deposited on a glassy carbon electrode (GCE) for Cu^2+^ detection via DPASV (Gong et al., 2023).

AuNPs are widely used in IIP-based sensors because they combine electrical conductivity, chemical stability, and surface plasmon resonance (SPR) properties. In electrochemical systems, AuNPs act as nanoscale electron-transfer mediators that mitigate the insulating character of imprinted polymer films, whereas in optical systems, they enhance signal output through plasmonic amplification (Karnwal et al., 2024). In one example, a surface ion-imprinted Cu^2+^ sensor employed AuNPs as conductive nanocarriers that confined imprinting to an ultrathin interfacial region while enabling efficient electron exchange during target binding (Gong et al., 2023). AuNPs have also been incorporated into SPR-based Cd^2+^ sensors (Figure 7), where nanoparticles embedded within the imprinted layer enhanced plasmonic coupling between the polymer and gold substrate, amplifying refractive-index changes associated with metal-ion rebinding (Bakhshpour and Denizli, 2020). This approach improved sensitivity while maintaining imprint-derived selectivity in wastewater samples.

Morphology-engineered AuNPs can further enhance performance. For example, flower-like AuNPs provided increased electroactive area and electrolyte accessibility for improved As^3+^ detection (Yang et al., 2023). However, nanoparticle aggregation and interfacial degradation remain important limitations during long-term operation in natural waters.

### 5.3 Carbon nanotubes and quantum dots

Carbon nanotubes (CNTs), particularly multi-walled carbon nanotubes (MWCNTs), provide one-dimensional conductive pathways that efficiently couple polymer recognition events to electrochemical readout. Their high aspect ratio and large accessible surface area make them effective conductive scaffolds for IIP films (Jose et al., 2023). In Pb^2+^ sensors, MWCNTs frameworks serve as percolated conductive backbones that accelerate electron exchange between imprinted binding sites and the electrode, improving voltammetric sensitivity in aqueous media (Sebastian and Mathew, 2018). Similar effects have been reported in Hg^2+^ detection systems combining MWCNTs with Hg^2+^-imprinted polymer nanoparticles (Alizadeh et al., 2018). Here, CNTs compensate for the insulating nature of polymer particles and improve their dispersion, shortening diffusion paths and enhancing uptake kinetics. Functionalized CNTs have also been applied to Ni^2+^ sensing, where improved hydrophilicity and dispersion stability increased accessibility of recognition sites (Aravind and Mathew, 2019). Despite these advantages, CNT-based composites may degrade through nanotube bundling, surface fouling, or gradual loss of polymer-CNT adhesion during prolonged exposure to water (Ardalani et al., 2020).

On the other hand, quantum dots (QDs) provide a different function by introducing size-dependent optical properties and efficient photoinduced charge-transfer mechanisms (Chen et al., 2019). In fluorescence-based IIP sensors, QDs often serve as the main signal transducers, while the imprinted polymer supplies selectivity. For example, Mn-doped ZnS quantum dots were integrated into a Cr^6+^-imprinted system where target binding produced fluorescence quenching through electron transfer between the analyte and the QD surface (Zhang et al., 2019). Encapsulation of the QDs within a thin imprinted shell preserved access to binding sites while maintaining strong optical output. Similarly, graphene quantum dots (GQDs) have also been incorporated into electrochemical IIP sensors for Hg^2+^ detection, where they act as conductive nanofillers that improve electron transfer and cavity accessibility (Zhang et al., 2019). However, QD-based systems remain limited by photostability, aggregation, and the challenge of maintaining uniform dispersion within polymer matrices.

### 5.4 Other nanomaterials

Beyond nanocarbons and noble metals, several additional nanomaterials have been explored to address specific limitations of polymer-based IIP sensors. Magnetic core-shell nanoparticles such as Fe_3_O_4_@SiO_2_ provide robust supports for imprinting while enabling magnetic manipulation of the sensing material (Chae et al., 2016). In Cd^2+^ sensing systems, these particles improved the exposure of imprinted cavities and facilitated integration into fiber-optic platforms (Shen et al., 2024).

For example, graphitic carbon nitride (g-C_3_N_4_) nanosheets offer a nitrogen-rich layered structure that promotes charge transfer and enhances interactions with hydrated metal ions (Dong et al., 2022). When incorporated into Hg^2+^-imprinted polymers, g-C_3_N_4_ improved electrochemical response through conductive domain formation within the composite matrix (Ganjali et al., 2019). Zinc oxide (ZnO) nanorods provide vertically aligned semiconducting architectures that support rapid ionic transport toward imprinted binding sites (Raub et al., 2024), although their chemical stability under acidic conditions remains a concern (Ait-Touchente et al., 2020). Whereas nanoporous gold structures provide bicontinuous metallic networks with large electroactive areas that facilitate charge transfer beneath imprinted layers, but structural coarsening during long-term use may reduce effective surface area (Ahmad and Bhattacharya, 2019; Islam et al., 2023).

### 5.5 Multi-nanomaterial architectures

Recent studies increasingly combine multiple nanomaterials within a single sensing architecture to exploit complementary functions such as conductivity enhancement, surface-area expansion, and catalytic amplification (Fu et al., 2025; Wu et al., 2020a; Wu et al., 2020b; Zhang et al., 2026). A graphite oxide/MWCNTs composite integrated with an IIP has been used for potentiometric Cu^2+^ detection, where graphite oxide improved hydrophilicity while MWCNTs provided conductive pathways for signal transduction (Topcu et al., 2018). Similarly, chitosan-based IIP sensors incorporating graphene sheets and AuNPs demonstrated how carbon nanostructures and metallic nanoparticles can jointly enhance electron transfer and electroactive area (Wu et al., 2020a).

**FIGURE 8:**
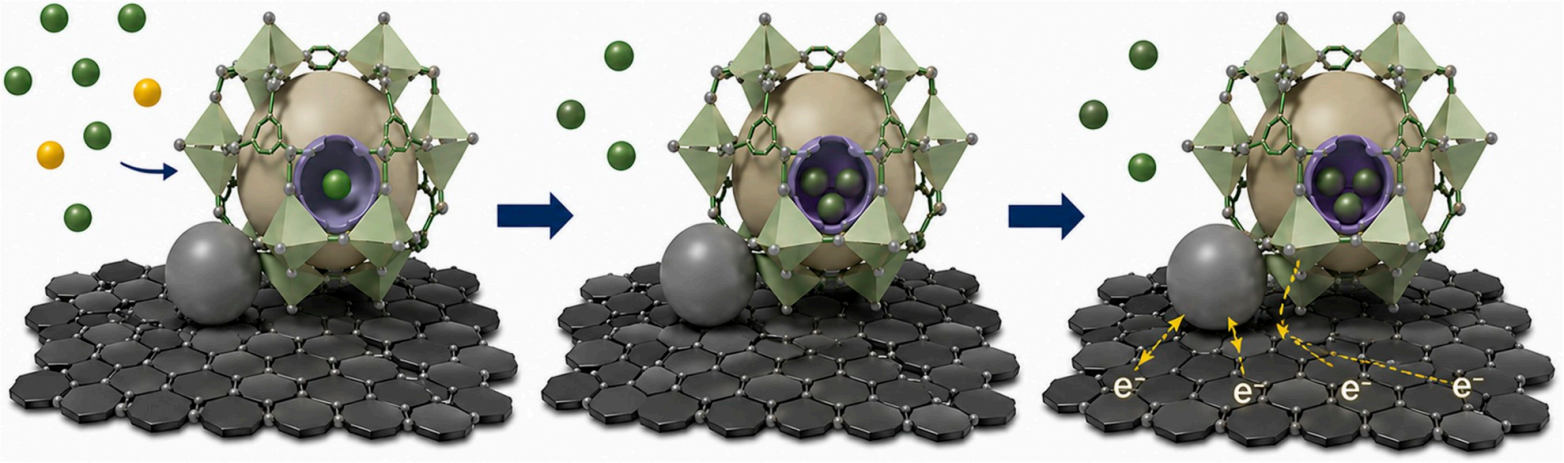
Working principle of an Ag@MOF–IIP/rGO hybrid sensor. The porous Ag@MOF nanohybrid concentrates target analytes near ion-imprinted recognition cavities, where selective binding occurs. Conductive Ag nanoparticles promote electron transfer to the underlying rGO network, resulting in an amplified electrical response for sensitive and selective analyte detection.

More complex systems combine MWCNTs, graphene, and AuNPs within a single composite matrix for Pb^2+^ detection, where carbon nanomaterials form a conductive network and AuNPs act as electron-transfer bridges (Wu et al., 2020b). Emerging hybrid designs also integrate metal-organic frameworks (MOFs) with conductive nanoparticles, such as Ag@MOF nanohybrids (Figure 8, to create porous interfaces that concentrate analytes near imprinted cavities while facilitating electron transfer (Fu et al., 2025). Another example is the incorporation of MoS_2_@Fe_3_O_4_ nanocomposites into IIP layers for Cd^2+^ sensing, where MoS_2_ provides large surface area and Fe_3_O_4_ enhances charge transport (Zhang et al., 2026). While these multi-nanomaterial systems often improve sensitivity and response kinetics, their performance depends on stable dispersion, robust interfacial bonding, and resistance to degradation during long-term operation in aqueous environments.

The contribution of nanomaterials to IIP sensor performance is mainly related to signal amplification, improved mass transport, and enhanced interfacial conductivity rather than direct chemical selectivity. The selectivity is primarily provided by the imprinted cavities, while nanomaterials improve how efficiently the binding event is translated into an analytical signal. Carbon-based nanomaterials such as graphene, rGO, and MWCNTs facilitate electron transfer and reduce the resistance of polymer films, which is particularly important because many IIP layers are intrinsically insulating. Metallic nanostructures such as AuNPs increase electroactive surface area and can provide electrocatalytic or plasmonic amplification. Semiconductor nanomaterials and quantum dots introduce optical or fluorescence transduction pathways, while magnetic and porous support can improve site accessibility and material handling. However, the benefit of nanomaterials depends strongly on their dispersion, loading amount, compatibility with the polymer matrix, and stability in water. Excessive nanomaterial loading may block recognition sites, increase nonspecific adsorption, or reduce reproducibility. Therefore, high-performance IIP sensors require an optimized balance between imprint-derived selectivity and nanomaterial-assisted signal enhancement (Ait Lahcen et al., 2023; Lahcen and Amine, 2019; Rowley-Neale et al., 2018; Yu et al., 2022).

## 6 Analytical performance and real-water validation of IIP-Based sensors

### 6.1 Analytical performance across sensing platforms

Table 2 summarizes the analytical performance of representative IIP-based sensors for toxic metal-ion monitoring in water. Across the reported studies, IIPs consistently enable selective recognition of target ions while achieving low detection limits, wide linear ranges, and acceptable recoveries in environmental samples. However, analytical performance varies significantly across sensing platforms and device architectures. These differences arise not only from the transduction mechanism but also from the imprinting chemistry, the accessibility of binding sites in aqueous media, and the extent to which nanomaterials enhance charge transfer and mass transport within the sensing interface.

**TABLE 2:** Analytical performance of representative IIP-based sensors for toxic metal-ion detection in water.

Target ion	Platform/Method	Nanomaterial	LOD (M)	Linear range (M)	Response time	Real water matrix	Recovery (%)	Performance assessment	Ref.
Ag^+^	Electrochemical (DPSV) CPE/IIP	MWCNTs	1.2 × 10^−10^	5.0 × 10−10–2.8 × 10^−7^	3–4 min	Tap, surface, groundwater	97–101	Very low LOD; rapid response; good recovery; validated in several matrices	Zhiani et al. (2016)
Hg^2+^	Electrochemical (SWASV) GCE/IIP	rGO	9.97 × 10^−11^	3.49 × 10−10–3.99 × 10^−7^	∼14 min	Tap, river, wastewater	97–103	Very low LOD; good recovery; validated in complex wastewater matrix	Ghanei-Motlagh et al. (2016)
Cd^2+^	Electrochemical (DPASV) CPE/IIP	—	1.33 × 10^−9^	4.45 × 10-9–3.56 × 10^−7^	∼6 min	River, dam, tap	97–102	Low LOD; good recovery; validated in multiple water matrices	Ghanei-Motlagh and Taher (2017)
Cd^2+^	Optical colorimetric PAD/IIP	—	3.56 × 10^−9^	8.90 × 10-9–8.90 × 10^−7^	∼12 min	Tap, lake, river	88–109	Portable optical format; acceptable but wider recovery range suggests matrix effects	Huang et al. (2017)
Pb^2+^	Electrochemical (DPV) CPE/IIP	—	1.3 × 10^−1^	1.0 × 10-9–7.5 × 10^−7^	2–3 min	Tap, river	99–102	LOD appears inconsistent with linear range; verify original value before submission	Luo et al. (2017)
Pb^2+^	Electrochemical (DPASV) GCE/IIP	MWCNTs	7.72 × 10^−10^	2.41 × 10-9–1.06 × 10^−7^	∼21 min	Tap, mineral	95–103	Low LOD; good recovery; longer response time than several electrochemical systems	Tarley et al. (2017)
Cu^2+^	Potentiometric electrode	GO/MWCNTs	4.0 × 10^−7^	1.0 × 10-6–1.0 × 10^−1^	∼3 s	Tap, river	102–104	Very fast response; broad linear range; higher LOD than stripping-based sensors	Topcu et al. (2018)
Ni^2+^	Electrochemical (DPV) Pt/IIP	MWCNTs	2.8 × 10^−8^	1.70 × 10-5–8.52 × 10^−5^	NR	Industrial wastewater	99–99.5	Validated in industrial wastewater; benchmarking limited because response time is not reported	Aravind and Mathew (2019)
Cr^6+^	Fluorescence sensor	ZnS:Mn QDs	1.05 × 10^−7^	3.85 × 10-7–1.92 × 10^−5^	NR	Tap, lake, wastewater	94–107	Optical platform with acceptable recovery; benchmarking limited by missing response time	Zhang et al. (2019)
As^3+^	Electrochemical (SWV) GCE/IIP	AuNPs	2.00 × 10−10	1.20 × 10−10–6.67 × 10−9	7–8 min	Drinking, river	81–113	Very low LOD; wider recovery range suggests stronger matrix influence	Yang et al. (2023)
Pb^2+^	Electrochemical (DPV) GCE/IIP	AuNPs/graphene/MWCNTs	2.8 × 10^−10^	1.0 × 10-9–1.0 × 10^−5^	∼10 min	Tap, river	97–105	Hybrid nanomaterial system; low LOD; broad working range; good recovery	Wu et al. (2020b)
Cd^2+^	Chemiresistive sensor IDE/IIP	rGO	7.38 × 10^−9^	1.78 × 10-8–1.78 × 10^−6^	∼5 min	Tap, lake, river	94–114	Portable electrical detection; rapid response; recovery indicates possible matrix variability	Hu et al. (2021)
Hg^2+^	QCM sensor	—	6.98 × 10^−8^	2.14 × 10-7–9.97 × 10^−6^	NR	Tap, wastewater	NR	Label-free detection; benchmarking is limited because response time and recovery are not reported	Low et al. (2024)
Cd^2+^	Optical interferometric sensor	Fe_3_O_4_	5.1 × 10^−11^	0–1.0 × 10^−6^	13–50 s	Aqueous samples	NR	Very low LOD and rapid response; real-matrix recovery not reported	Shen et al. (2024)

DPSV: differential pulse stripping voltammetry, CPE: carbon paste electrode, MWCNTs: multi-walled carbon nanotubes, SWASV: square wave anodic stripping voltammetry, rGO: reduced graphene oxide, DPASV: differential pulse anodic stripping voltammetry, PAD: Paper-based analytical device, DPV: differential pulse voltammetry, Pt: Platinum electrode, Fe_3_O_4_: magnetic nanoparticles, GO: graphite oxide, ZnS: zinc sulfide, QDs: Quantum dots, SWV: square wave voltammetry, AuNPs: gold nanoparticles, IDE: interdigitated au electrodes, QCM: quartz crystal microbalance, Ag^+^: silver(I) ion, As^3+^: arsenic (III) ion, Cd^2+^: cadmium (II) ion, Cr^3+^: chromium (III) ion, Cr^6+^: chromium (VI) ion, Cu^2+^: copper (II) ion, Hg^2+^: mercury (II) ion (mercuric ion), Ni^2+^: nickel (II) ion, Pb^2+^: lead (II) ion, NR: not reported., LOD: limit of detection.

A clear trend in Table 2 is the dominance of electrochemical sensing platforms. Techniques such as differential pulse voltammetry, stripping voltammetry, and potentiometric measurements appear most frequently, reflecting the strong compatibility between ion rebinding at the polymer-electrode interface and electrochemical signal generation. In these systems, metal-ion coordination within imprinted cavities perturbs the interfacial charge distribution or redox behavior, allowing highly sensitive detection through established electroanalytical methods.

For widely studied contaminants such as Cd^2+^, Pb^2+^, and Hg^2+^, the strongest analytical performance is typically observed in hybrid architectures combining selective imprinted polymers with conductive nanomaterials, including rGO, ERGO, MWCNTs, AuNPs, or magnetic nanostructures. These materials reduce charge-transfer resistance, expose a greater fraction of imprinted binding sites, and alleviate diffusion limitations associated with dense cross-linked polymer films. In contrast, polymer-only systems frequently exhibit higher detection LODs and slower response times, even when the imprinting chemistry is highly selective. These observations indicate that high-performance IIP sensors typically depend on the synergy between selective coordination chemistry and a conductive, nanostructured transducer interface that remains accessible in aqueous media.

Optical and fluorescence-based sensors also achieve environmentally relevant detection limits, particularly for Pb^2+^, Cd^2+^, Cr^6+^, and Ni^2+^. Their principal advantage lies in non-electrical readout and compatibility with portable or disposable sensing formats. However, compared with electrochemical sensors, optical systems often exhibit narrower working ranges, longer analysis times, or more limited validation in complex environmental matrices. Optical signals can also be affected by film thickness, background scattering, or matrix-dependent attenuation, which can reduce analytical robustness in natural waters.

For less frequently studied ions such as Ag^+^, As^3+^, Cr^3+^, and Ni^2+^, the available performance data remain relatively limited. In these cases, aqueous speciation, redox chemistry, hydrolysis, and strong hydration effects may exert greater influence on analytical performance than in the extensively studied Cd^2+^ and Pb^2+^ systems. Expanding sensor development toward these environmentally relevant metals will require greater attention to both imprinting chemistry and matrix-dependent metal speciation.

The main factors affecting analytical performance can be grouped into four categories: imprinting-related, transport-related, transducer-related, and matrix-related factors. Imprinting-related factors include ligand type, cavity fidelity, template-removal efficiency, binding-site density, and the strength of metal–ligand coordination. Transport-related factors include polymer thickness, porosity, swelling behavior, and accessibility of hydrated ions to the recognition sites. Transducer-related factors include electrode conductivity, optical signal stability, piezoelectric mass sensitivity, and the efficiency of signal amplification. Matrix-related factors include pH, ionic strength, dissolved organic matter, competing ions, suspended particles, and fouling species. These factors explain why sensors designed for the same target ion can show different detection limits, response times, linear ranges, and recoveries. Therefore, analytical performance should be interpreted as the combined outcome of imprinting chemistry, material architecture, transducer design, and environmental matrix (Sala et al., 2022; Tchekwagep et al., 2022).

A broader conclusion from Table 2 is that direct comparison between studies remains challenging because analytical conditions and reporting practices are not standardized. Parameters such as response time, operational stability, regeneration cycles, and selectivity testing are reported inconsistently, and real-sample validation frequently relies on spiked matrices rather than blind environmental samples. In addition, many studies do not clearly distinguish between total metal concentration and the free-ion fraction that is actually recognized by imprinted binding sites in aqueous solution. Consequently, although IIP-based sensors can achieve LODs approaching regulatory requirements for several priority toxic metal ions, more uniform benchmarking protocols will be necessary to enable meaningful cross-platform comparisons and guide further technological development. Overall, electrochemical IIP sensors currently provide the best balance of sensitivity, speed, and matrix compatibility, whereas optical and piezoelectric formats offer complementary advantages in portability, label-free operation, or simplified readout.

Comparison across sensing platforms shows that electrochemical IIP sensors generally provide the strongest analytical performance for trace toxic metal-ion detection because they combine selective ion rebinding with sensitive current-, potential-, or impedance-based readout. Hybrid electrochemical systems containing conductive nanomaterials frequently achieve lower LODs than polymer-only sensors because nanomaterials improve electron transfer, increase electroactive area, and reduce diffusion limitations. Optical and fluorescence-based IIP sensors also achieve environmentally relevant LODs and offer advantages in simple readout and portability, but their performance may be more sensitive to optical background, turbidity, and matrix-induced quenching. Piezoelectric sensors provide label-free detection, but their use remains more limited because frequency responses depend strongly on film adhesion, swelling behavior, and mechanical stability. Overall, the data indicates that electrochemical platforms are currently the most mature for quantitative detection, while optical and piezoelectric formats remain valuable complementary approaches for simplified or label-free monitoring (Sala et al., 2022; Sulthana et al., 2024; Tchekwagep et al., 2022).

### 6.2 Validation in real water matrices

While analytical metrics provide an indication of intrinsic sensor performance, validation in real water matrices is essential for assessing practical applicability. Natural waters contain variable ionic strength, hardness ions, dissolved organic matter (DOM), suspended particulates, and competing trace metals that influence metal speciation and sensor response. These matrix components may reduce the free-ion fraction available for rebinding at imprinted cavities or block access to recognition sites through nonspecific adsorption. As a result, successful validation in real samples provides both analytical confirmation and an indication that the coordination chemistry and structural accessibility of the imprinted cavities remain effective under environmentally relevant conditions (Abdul Halim et al., 2024; Ait Lahcen et al., 2023; Hande et al., 2015; Metwally et al., 2021; Rebelo et al., 2021; Sala et al., 2022; Tchekwagep et al., 2022).

Table 3 provides a practical comparison of IIP-based sensors by linking real-water validation with LOD, recovery, matrix effects, and sensor applicability. Most reported platforms showed recoveries within or close to the acceptable analytical range, confirming the applicability of IIP-based sensors in diverse water samples, including tap, mineral, river, lake, wastewater, and industrial wastewater. However, wider recovery ranges, such as 81%–113% for As^3+^ and 88%–109% for colorimetric Cd^2+^ detection, indicate stronger matrix effects and the need for careful real-sample validation. Matrix components such as dissolved organic matter, competing ions, salinity, chloride complexation, turbidity, fouling, optical background, and fluorescence quenching can affect free-ion availability and signal stability. Overall, electrochemical IIP sensors appear particularly suitable for quantitative monitoring, while optical, fluorescence, and paper-based platforms offer advantages for portable or multiplexed screening but require careful control of matrix-related interferences.

**TABLE 3:** Real-water validation and matrix effects of representative IIP-based toxic metal-ion sensors.

Metal ion	Platform	Validated matrices	LOD (M)	Recovery (%)	Reported/Expected matrix effects	Practical interpretation	Ref
Ag^+^	Electrochemical DPSV CPE/IIP with MWCNTs	Tap, surface, groundwater	1.2 × 10^−10^	97–101	Low-to-moderate matrix complexity	Suitable for routine water screening	Zhiani et al. (2016)
Hg^2+^	Electrochemical SWASV GCE/IIP with rGO	Tap, river, wastewater	9.97 × 10^−11^	97–103	Organic matter and competing ions may affect the response	Promising for polluted water monitoring	Ghanei-Motlagh et al. (2016)
Cd^2+^	Electrochemical DPASV CPE/IIP	River, dam, tap	1.33 × 10^−9^	97–102	Moderate matrix influence expected in natural waters	Applicable to routine Cd^2+^ monitoring	Ghanei-Motlagh and Taher (2017)
Cd^2+^	Optical colorimetric PAD/IIP	Tap, lake, river	3.56 × 10^−9^	88–109	Optical background and water composition may affect colorimetric response	Useful for portable screening	Huang et al. (2017)
Pb^2+^	Electrochemical DPASV GCE/IIP with MWCNTs	Tap, mineral water	7.72 × 10^−10^	95–103	Moderate matrix influence in potable water	Good applicability in drinking-water analysis	Tarley et al. (2017)
Cu^2+^	Potentiometric GO/MWCNT electrode	Tap, river	4.0 × 10^−7^	102–104	Competing divalent ions may influence the potentiometric response	Rapid detection, but selectivity must be verified	Topcu et al. (2018)
Ni^2+^	Electrochemical DPV Pt/IIP with MWCNTs	Lake and industrial wastewater	2.8 × 10^−8^	99–99.5	Complex wastewater composition may affect ion availability	Relevant for industrial effluent monitoring	Aravind and Mathew (2019)
Cr^3+^	DPV Pt/MWCNT-IIP	Industrial wastewater	5.0 × 10^−8^	NR	Cr^3+^ hydrolysis near neutral pH and complex industrial-wastewater composition may affect free-ion availability	Useful for Cr^3+^ sensing/extraction in industrial wastewater, but pH/speciation control is important	Aravind and Mathew (2018)
Cr^6+^	Fluorescence sensor with ZnS:Mn QDs	Tap, lake, wastewater	1.05 × 10^−7^	94–107	Fluorescence may be affected by quenching and background signal	Suitable when optical matrix effects are controlled	Zhang et al. (2019)
As^3+^	Electrochemical SWV GCE/IIP with AuNPs	Drinking, river	2.00 × 10^−10^	81–113	Wider recovery range indicates stronger matrix effects	Requires careful matrix validation	Yang et al. (2023)
Cd^2+^/Pb^2+^	Fluorescence QD-based IIP	Surface water, drinking water, seawater	2.18 × 10−9/1.62 × 10^−9^	NR	Salinity and optical background may affect fluorescence	Demonstrates multi-metal detection feasibility	Zhou et al. (2020)
Cd^2+^/Cu^2+^/Hg^2+^/Pb^2+^	Paper-based microfluidic fluorescence device	Seawater	3.49 × 10−11–2.36 × 10^−10^	NR	High ionic strength and chloride complexation are critical	Useful for complex saline waters if validated	Wang et al. (2021b)

LOD: limit of detection, DPSV: differential pulse stripping voltammetr, SWASV: square-wave anodic stripping voltammetry, DPASV: differential pulse anodic stripping voltammetry, SWV: square-wave voltammetry, DPV: differential pulse voltammetry, CPE: carbon paste electrode, GCE: glassy carbon electrode, Pt: platinum, IIP: ion-imprinted polymer, MWCNTs: multi-walled carbon nanotubes, rGO: reduced graphene oxide, GO: graphene oxide, PAD: paper-based analytical device, QDs/QD: quantum dots/quantum dot, AuNPs: gold nanoparticles, NR: not reported, Ag^+^: silver(I) ion, As^3+^: arsenic (III) ion, Cd^2+^: cadmium (II) ion, Cr^6+^: chromium (VI) ion, Cr^3+^: chromium (III), Cu^2+^: copper (II) ion, Hg^2+^: mercury (II) ion, Ni^2+^: nickel (II) ion, Pb^2+^: lead (II) ion.

### 6.3 Drinking and surface waters

Tap and drinking waters represent the most common validation matrices for IIP sensors because their moderate ionic composition provides a practical intermediate between laboratory buffers and more chemically complex environmental waters. Several studies demonstrate that electrochemical IIP sensors can maintain selective recognition of target ions under these conditions. For example, Pb^2+^ determination in tap and mineral water using composite IIP electrodes shows strong selectivity against common divalent cations and satisfactory recoveries, indicating that the imprinted coordination cavities remain stable in typical potable-water chemistries (René et al., 2020; Shamsipur et al., 2018; Wu et al., 2020b). Similarly, Hg^2+^ sensors employing carbon-paste or nanocarbon-modified electrodes have demonstrated trace-level sensitivity in tap-water samples (Fadillah et al., 2022).

Surface waters introduce additional complexity due to dissolved organic matter, suspended particles, and fluctuating redox conditions that influence metal speciation and bioavailability. Organic ligands present in DOM can strongly complex metals such as Cd^2+^ and Pb^2+^, reducing the free-ion concentration available for binding to imprinted sites. Studies using graphene-assisted electrochemical IIP sensors for Cd^2+^ detection in river and lake samples illustrate how conductive nanostructures can partially compensate for these challenges by improving mass transport and maintaining access to surface-imprinted binding sites (Wang et al., 2020; Wang et al., 2023). Nevertheless, practical measurements often require pH adjustment or electrode cleaning to mitigate fouling and maintain reproducible signals.

### 6.4 Seawater and industrial wastewater

Seawater and industrial effluents represent the most chemically demanding environments for IIP sensors. In seawater, high ionic strength and elevated chloride concentrations intensify competitive binding and destabilize electrochemical baselines. Under such conditions, both the accessibility of the imprinted sites and the conductivity of the sensing interface become critical. One example is a microfluidic paper-based analytical device capable of simultaneously detecting Cu^2+^, Cd^2+^, Pb^2+^, and Hg^2+^ in coastal seawater using integrated on-chip enrichment (Wang et al., 2021b). This approach demonstrates how imprinting selectivity can be combined with microfluidic sample handling to maintain sensitivity in highly saline environments.

Industrial wastewater and effluents introduce additional analytical challenges because they may contain high concentrations of organic contaminants, extreme pH conditions, and mixtures of multiple toxic metal ions that compete for coordination sites within the polymer network. Detection of Cr^3+^ in electroplating wastewater illustrates that carefully designed imprinted interfaces can retain selective binding in strongly competitive environments when solution speciation and pH conditions are properly controlled (Aravind and Mathew, 2018; Hu et al., 2024). Similarly, Hg^2+^ sensors validated in industrial waters, often involving diluted or pretreated samples highlight the importance of regeneration procedures and sample preparation strategies for maintaining stable analytical signals in fouling-prone matrices (Low et al., 2024).

### 6.5 Overall assessment of matrix effects

Across the studies summarized in Tables 2, 3, a consistent trend emerges: the analytical performance of IIP-based sensors is strongly influenced by the chemical environment in which they operate. In relatively simple matrices such as drinking water, sensor performance is largely governed by the intrinsic selectivity of the imprinted coordination sites. As matrix complexity increases toward surface waters, seawater, and industrial effluents, additional factors, including metal speciation, ionic competition, surface fouling, and signal stability, become increasingly important.

Successful strategies for maintaining analytical performance in complex waters include surface imprinting, conductive nanomaterial scaffolds, antifouling interfaces, and integrated enrichment or regeneration protocols. However, many studies still rely primarily on buffer solutions or spiked matrices rather than fully representative environmental samples. Greater emphasis on rigorous real-matrix validation and standardized performance reporting will therefore be essential for translating promising laboratory-scale IIP sensors into reliable field-deployable technologies for environmental toxic metal-ion monitoring (Abdul Halim et al., 2024; Ait Lahcen et al., 2023; Hande et al., 2015; Metwally et al., 2021; Rebelo et al., 2021; Sala et al., 2022; Tchekwagep et al., 2022).

## 7 Perspectives: advantages, limitations, and future directions

IIP sensors provide a chemically rational platform for selective toxic metal-ion monitoring because imprinting generates binding cavities whose geometry, charge distribution, and coordination environment are complementary to the template ion. These sites mimic metal-ligand interactions in solutions and enable selective recognition even in the presence of competing species, addressing a key limitation of purely electrochemical detection based only on potential control (Malitesta et al., 2017). When coupled with electrochemical transduction, particularly stripping voltammetry, impedance spectroscopy, or potentiometry, the imprinted layer can also function as a selective enrichment interface, enabling trace and sub-trace detection with portable platforms (Metwally et al., 2021; Sala et al., 2022).

A major advantage of IIP systems is their chemical robustness and architectural versatility. Unlike biological receptors, imprinted polymers generally tolerate broader pH ranges and harsher regeneration conditions while maintaining structural integrit (Malitesta et al., 2017; Tchekwagep et al., 2022). Their chemistry is adaptable to particle-based composites, drop-cast layers, and electropolymerized thin films, and performance is often enhanced by hybridization with conductive nanomaterials that improve charge transfer and mass transport without replacing imprint-derived selectivity (Ait Lahcen et al., 2023; Metwally et al., 2021). These features explain why many IIP sensors show acceptable recoveries in tap water, river water, seawater, and industrial wastewater (Mukendi et al., 2025; Tchekwagep et al., 2022).

Despite these advantages, several limitations still restrict broader deployment. Recognition-site instability can arise when coordinating ligands are physically entrapped rather than covalently incorporated, leading to ligand leaching and gradual loss of selectivity during template removal or repeated operation (Abdul Halim et al., 2024; Sala et al., 2022). Dense or highly cross-linked films can also bury binding sites and slow diffusion of hydrated ions, whereas excessively thick coatings reintroduce mass-transfer limitations even in surface-imprinted formats (Metwally et al., 2021). In addition, many imprinted polymers are intrinsically nonconductive, so electrochemical performance often depends on conductive additives whose benefits vary with porosity, hydrophilicity, dispersion quality, and coupling to the electrode surface (Sala et al., 2022; Zarejousheghani et al., 2021). Fabrication reproducibility remains another challenge because polymerization conditions, template extraction efficiency, and film deposition strongly affect site density, accessibility, and interfacial stability (Ait Lahcen et al., 2023; Mukendi et al., 2025).

Future development of IIP-based toxic metal-ion sensors should focus on improving the relationship between recognition chemistry, interfacial architecture, and real-sample performance. From a mechanistic perspective, stronger and more selective coordination motifs, including multidentate or mixed-donor monomers, could improve cavity fidelity and binding affinity for target ions. From a structural perspective, surface-imprinted layers, ultrathin electropolymerized films, and porous nanostructured supports can increase the accessibility of recognition sites and shorten diffusion pathways. From a transduction perspective, conductive and optically active nanomaterials should be optimized to enhance signal generation without blocking imprinted cavities or increasing nonspecific adsorption. In addition, future studies should place greater emphasis on reproducibility, regeneration, long-term stability, antifouling behavior, and validation in authentic environmental samples rather than only spiked laboratory matrices. Standardized benchmarking against conventional methods such as ICP-MS, ICP-OES, or AAS will also be essential to define the practical role of IIP sensors as complementary tools for rapid screening and field monitoring (Ait Lahcen et al., 2023; Garciá-Miranda Ferrari et al., 2020; Metwally et al., 2021; Sala et al., 2022).

Also, future progress will require advances in both polymer chemistry and device engineering. Stronger and more selective coordination motifs, including multidentate or mixed-donor monomers, may improve binding strength and cavity fidelity, while surface-imprinting and controlled electropolymerization can enhance accessibility and reproducibility. Greater emphasis is also needed on realistic validation and standardized benchmarking in authentic environmental waters, including long-term durability, regeneration, fouling resistance, and comparison with reference analytical methods. With continued progress in imprinting chemistry, interfacial design, and validation rigor, IIP-based sensors are well-positioned to complement established laboratory techniques and support rapid, on-site water-quality monitoring in environmental and industrial settings. As water-quality monitoring increasingly shifts toward decentralized sensing and real-time environmental surveillance, imprint-based recognition strategies may play an important role in bridging laboratory analytical chemistry with deployable field diagnostics.

## 8 Conclusion

This review highlights the principles of IIPs and their application in sensing platforms for toxic metal-ion detection in water. Imprinting creates binding cavities whose coordination geometry, charge distribution, and size complement the target ion, enabling selective recognition even in the presence of competing species. Among reported systems, electrochemical IIP sensors dominate because they effectively combine selective metal uptake with sensitive signal transduction. Voltammetric platforms particularly benefit from the preconcentration capability of imprinted layers, while conductive nanomaterials often enhance electron transfer and mass transport, enabling trace-level detection in diverse water matrices, including tap water, surface waters, seawater, and industrial effluents. Optical, fluorescence, and piezoelectric approaches further demonstrate the versatility of imprint-based recognition, though they may be more susceptible to matrix effects such as turbidity and surface fouling.

This coordination-defined recognition is central to IIP performance because the choice of functional monomer, donor atoms, ligand denticity, and template–monomer complex stability determines the fidelity, accessibility, and reversibility of the final binding sites.

Despite these advances, several challenges still limit widespread environmental deployment. Diffusion constraints within dense polymer networks can reduce site accessibility, while incomplete template removal or weak ligand immobilization may affect long-term selectivity and reproducibility. Environmental matrices containing competing ions, dissolved organic matter, and fouling species can further complicate sensor performance. Addressing these issues will require improved imprinting chemistries, surface-imprinted or ultrathin films with controlled thickness, and antifouling interface designs. Equally important is the adoption of standardized benchmarking and validation of analytical methods in authentic environmental waters against established standards. With continued advances in polymer chemistry, interfacial engineering, and portable sensing technologies, IIP-based sensors are well-positioned to complement conventional laboratory techniques and enable reliable on-site monitoring of water quality.
